# Classification and Quality Evaluation of Tobacco Leaves Based on Image Processing and Fuzzy Comprehensive Evaluation

**DOI:** 10.3390/s110302369

**Published:** 2011-02-25

**Authors:** Fan Zhang, Xinhong Zhang

**Affiliations:** 1 Institute of Image Processing and Pattern Recognition, Henan University, Kaifeng 475001, China; 2 College of Computer and Information Engineering, Henan University, Kaifeng, 475001, China; 3 Computing Center, Henan University, Kaifeng 475001, China

**Keywords:** image analysis, tobacco leaf, fuzzy sets, fuzzy comprehensive evaluation, artificial neural network

## Abstract

Most of classification, quality evaluation or grading of the flue-cured tobacco leaves are manually operated, which relies on the judgmental experience of experts, and inevitably limited by personal, physical and environmental factors. The classification and the quality evaluation are therefore subjective and experientially based. In this paper, an automatic classification method of tobacco leaves based on the digital image processing and the fuzzy sets theory is presented. A grading system based on image processing techniques was developed for automatically inspecting and grading flue-cured tobacco leaves. This system uses machine vision for the extraction and analysis of color, size, shape and surface texture. Fuzzy comprehensive evaluation provides a high level of confidence in decision making based on the fuzzy logic. The neural network is used to estimate and forecast the membership function of the features of tobacco leaves in the fuzzy sets. The experimental results of the two-level fuzzy comprehensive evaluation (FCE) show that the accuracy rate of classification is about 94% for the trained tobacco leaves, and the accuracy rate of the non-trained tobacco leaves is about 72%. We believe that the fuzzy comprehensive evaluation is a viable way for the automatic classification and quality evaluation of the tobacco leaves.

## 1. Introduction

Because of the diversity and complexity of tobacco leaves, most of the classification and the quality evaluation of the flue-cured tobacco leaves are manually operated. It is a rigorous task. The tobacco leaves must be carefully classified by size, texture and color, all aided by a well-seasoned expert’s feeling about the fine properties of the leaves. Errors often occur when the experts are tired, and the results of classification and quality evaluation relies on the judgmental experience of experts and many other factors, such as the emotion of experts, the human eyesight, the condition of illumination, *etc*. The grading process is extremely laborious, making the classification and the quality evaluation subjective and experientially based, while the efficiency and the stability of error rate are not satisfying enough. New technology and equipments are needed to automate the quality inspection process of tobacco leaves.

The criterion of the quality evaluation of flue-cured tobacco leaves usually include color, size, shape and disfigurement of flue-cured tobacco leaf, *etc*. Most of these properties relate to the human vision. Recently, image processing, machine vision, pattern recognition and fuzzy mathematics make a rapid progress with the development of the computer and multimedia technologies. The automatic classification method of tobacco leaves is likely to be enabled by these technologies. Some works on application of image processing to extract the features of tobacco leaves and works on the tobacco leaves classification have been presented. Zhang *et al.* presented a transformation technique from RGB signals to the Munsell system for the color analysis of tobacco leaves. Zhang *et al*. and Garcia *et al*. [1–3] introduced the development of a virtual expert for the classification of tobacco leaves. Zhang *et al*. [4] proposed an algorithm that extract the features of tobacco leaves based on neural network. There are also some other works that have been reported [5–8].

Fuzziness seems to pervade most human perception and thinking processes. It is the argument of this study, therefore, that the theory of fuzzy sets is highly suitable to the tasks of the classification and the quality evaluation of flue-cured tobacco leaves. In this paper, fuzzy comprehensive evaluation is used to classify flue-cured tobacco leaves. The aim of this study is to utilize the theory of fuzzy sets to demonstrate the applicability of fuzzy logic for grading of tobacco leaves.

The rest of this paper is organized as follows. Section 2 is a brief introduction about the mechanics of grading process of flue-cured tobacco leaves. In Section 3, a grading system based on image processing techniques is introduced, which automatically inspects and evaluates quality of flue-cured tobacco leaves. After we get the images of tobacco leaves from the image processing system, the features extraction of tobacco leaves is introduced in Section 4. In Section 5, we briefly introduce theory of fuzzy set and fuzzy comprehensive evaluation. Automatic classification of tobacco leaves is discussed in Section 6. The experimental results are shown in Section 7. The conclusions of this paper are drawn in Section 8.

## 2. Tobacco Leaf Grading

The quality inspection of tobacco leaves consists of two main aspects: internal and external examinations. The internal quality inspection is usually achieved by human sensory, smoking test or chemical analysis, while the external quality inspection is mainly achieved through human vision. It is costly and yet time-consuming to inspect internal quality since tobacco leaves contain too many ingredients to be handled. As an alternative, external quality examination is often used instead in the examination of internal quality of tobacco leaves, since external features are closely related to internal quality. The external quality inspection of tobacco leaves includes judgment of color, maturity, surface texture, size and shape. Human vision, which is inevitably limited by personal, physical and environmental factors, has been the predominant means of inspection.

The detailed grading standards of flue-cured tobacco leaves may vary from one country or even one tobacco strain to another, but the general method and the concerned external features of tobacco leaves are quite similar [9,10].

**Figure 1.**
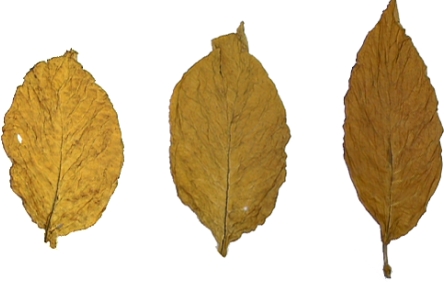
Three images of tobacco leaves which are obtained from image processing system of tobacco leaves grading.

Tobacco leaves are classified as normal or abnormal leaves. Since the normal leaves are more common than the abnormal ones, they are discussed in this paper. The normal tobacco leaves are graded based on the position they grow on the stalk and the color they have. Three position categories (so-called large groups) are identified, that is, lugs (X), cutters (C) and leaf (B), corresponding to lower, middle and upper portion of a stalk. The judgment for growth position is mainly based on the color, surface texture, shape and vein condition of the tobacco leaves. Three hue categories (so-called small groups), lemon (L), orange (F) and red-brown (R), are specified. The groups are formed by combining the position and color categories, that is, lugs lemon (XL), lugs orange (XF); cutters lemon (CL), cutters orange (CF); leaf lemon (BL), leaf orange (BF) and leaf red-brown (BR). Each group is further divided into 3 or 4 grades based on the chroma, hue uniformity across the whole leaf, maturity (mainly judged from color), size and surface texture. The classification of tobacco leaves is symbolized as, for example, X2L, where X indicates that the leaf is from the lugs position, L denotes that the leaf is in the hue category of lemon yellow, and the 2 means that the tobacco leaf is at the second grade of this group. Three flue-cured tobacco leaves are shown as Figure 1.

## 3. Image Processing System

**Figure 2.**
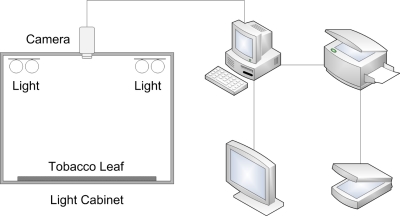
The image processing system of tobacco leaves grading.

An image processing system of tobacco leaves grading is constructed (Figure 2). Actually, the lighted cabinet is the same one as Zhang has used [1], but all other equipments, such as computer and camera, has been updated. The image processing system is consisted of a color camera (Panasonic WV-CP470 0.8 Lux 752 × 582 1/3 inch CCD), an OK-C20B PCI Bus Frame Grabber (Chinese Automation Ltd., Beijing) with 512×512 pixel resolution and 24-bit color, a monitor, and a computer. A lighted cabinet with 800 × 800 × 700 mm (length, width and height) is constructed to control illumination. The chamber is illuminated by two LED lights.

LED lights are fixed on the top of the cabinet. Tobacco leaf is placed on a piece of white board that could slide into the cabinet. The digital color image of tobacco leaf is obtained by the color camera and sent to computer, in which it is saved as a pixel matrix. The images is composed of three sets of pixels for each color. The image’s size is 512 × 512. Other equipments include a printer and a scanner. Delphi programming language is used to implement all algorithms.

## 4. Features Extraction

In this research, the digital image system process 24-bit color images of tobacco leaves. The size of tobacco leaves image is 512 × 512. Features of tobacco leaves can be extracted according to image processing, which include the shape, color, and texture features. These features are stored in a database in computer. The shape features include the surface area, surface perimeter and the disfigurement of tobacco leaf. The color features include the variance of red, green and blue channel of digital image of tobacco leaves. The texture features include the texture energy, texture entropy and texture contrast of digital image of tobacco leaves.

### 4.1. Color Features

Color is an important feature of tobacco leaves due to its close association with the perceived quality. It is a widely used parameter in the evaluation of their maturity, freshness, nutritional condition and growth factors.

There are a number of color systems, such as the RGB, CIE1931, HSI and Munsell [11], that can be used in machine vision for color evaluation. Each system has its own advantages and is used to satisfy different requirements. The color in most color systems is different from human color vision and cannot be intuitively understood. In order to a machine can simulate human color vision, the selected color system should possess the color spatial parameters and structure that are similar to human color sensation.

For the reason of economy in calculation, this research chooses the RGB color system to extract the color features of tobacco leaves. RGB images use three colors, red, green and blue to reproduce 16.7 million colors. Computer monitors display colors using this model. This research chooses three color features, they are the variance of red, green and blue channel of the digital image of tobacco leaves.

### 4.2. Shape Features

Image processing and machine vision techniques are used for the shape features extraction. Before the extraction of shape features, the edge data of the tobacco leaves should obtained firstly. Laplacian operator is used for the edge detection [12,13]. It is defined as follows,

(1) $$\nabla^{2}f\left(x,y\right)=\frac{\partial^{2}f\left(x,y\right)}{\partial^{2}x}+\frac{\partial^{2}f\left(x,y\right)}{\partial^{2}y}.$$

Laplacian operator is a quadratic derivative that is used to detect the abrupt zero-crossing of image intensity and often yields more exact edge detecting result. However, because of the presence of noises, the edge detection using Laplacian operator is not satisfying. This research chooses a Gaussian low-pass filter for the pre-smoothing. Gaussian low-pass filter is defined as follows,

(2) $$g\left(x,y\right)=G\left(x,y\right)*f\left(x,y\right)=\frac{1}{2\pi \sigma^{2}}\exp\left(-\frac{x^{2}+y^{2}}{2\sigma^{2}}\right)*f\left(x,y\right).$$

Laplacian operator and the Gaussian impulse response can be combined as a single Laplacian of Gaussian kernel,

(3) $$\nabla^{2}\frac{1}{2\pi \sigma^{2}}\exp\left(-\frac{x^{2}+y^{2}}{2\sigma^{2}}\right)=-\frac{1}{\pi \sigma^{4}}\left(1-\frac{x^{2}+y^{2}}{2\sigma^{2}}\right)\exp\left(-\frac{x^{2}+y^{2}}{2\sigma^{2}}\right).$$

**Figure 3.**
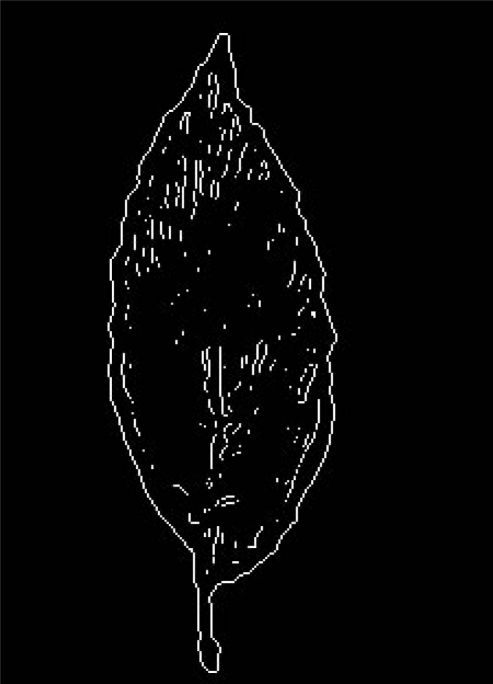
Edge detection of a tobacco leaf using Laplacian of Gaussian filtering.

24-bit color image of tobacco leaves are transformed to binary image. After Laplacian of Gaussian filtering, a closed and connected contour is obtained when interior points are eliminated. Edge detection of a tobacco leaf using Laplacian of Gaussian filtering is shown in Figure 3.

**Figure 4.**
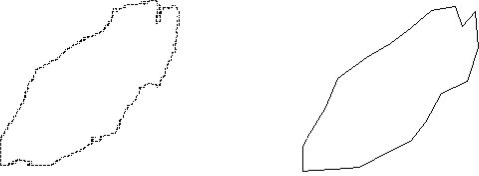
The polygonal fitting of edge of the tobacco leaf. The left image is the original tobacco leaf edge, the right image is the polygonal fitted edge.

The polygonal fitting is implemented to the edge curve when we get the edge data of the tobacco leaf. The aim of polygonal fitting is to substitute the smooth tobacco leaf edge to a polygonal edge. The polygonal fitting of edge of the tobacco leaf is shown in Figure 4. The vertexes in polygon is connected and formed some triangle. The sum number of pixels in each side of the fitted polygon is the approximate perimeter of the tobacco leaf. The hight, width and area of tobacco leaf can be calculated from the triangled polygon. The surface area of the fitted polygon is the approximate surface area of the tobacco leaf. We record the number of all the interior pixels of the fitted polygon, and record the number of all the interior pixels which color is same as the color of the background color. The ratio of two kinds of pixels is the disfigurement of the tobacco leaf.

### 4.3. Texture Features

The gray-level co-occurrence matrix (GLCM) is used for the extraction of texture features. The gray-level co-occurrence matrix can reveal certain properties about the spatial distribution of the gray-level in the texture image [14–16]. A gray-level co-occurrence matrix by calculating how often a pixel with the intensity (gray-level) value *i* occurs in a specific spatial relationship to a pixel with the value *j*. By default, the spatial relationship is defined as the pixel of interest and the pixel to its immediate right (horizontally adjacent), but we can specify other spatial relationships between the two pixels. Each element (*i, j*) in the resultant GLCM is simply the sum of the number of times that the pixel with value *i* occurred in the specified spatial relationship to a pixel with value *j* in the input image. For example, if most of the entries in the GLCM are concentrated along the diagonal, the texture is coarse with respect to the specified offset. The *N* × *N* co-occurrence matrix describes the spatial alignment and the spatial dependency of the different gray-level, whereas *N* is the number of gray-level in the image.

The dimension of gray-level co-occurrence matrix is same as the number of gray-level in images. In order to simplify calculation, firstly, we degrade the color image of tobacco leaf to a gray-level image. The maximal number of gray-level in this image is 256, so the maximal dimension of gray-level co-occurrence matrix is 256 × 256. But the 256 × 256 matrix is still too difficult to calculate, so we further degrade the 256 gray-level image to a 32 gray-level image, then the dimension of gray-level co-occurrence matrix is 32 × 32. During degrading the 256 gray-level image to 32 gray-level image, it will surely loss some subtle information in the image. This degradation is only used in the calculation of gray-level co-occurrence matrix, and will improve the computing efficiency greatly.

The gray-level co-occurrence matrix *p_δ_*(*i, j*) is defined as follows,

(4) $$p_{\delta }\left(i,j\right)=\left\{\left(x,y\right)|f\left(x,y\right)=i,\;\;f\left(x+\mathrm{DX},y+\mathrm{DY}\right)=j\right\}.$$

The entry (*i, j*) of *p_δ_*(*i, j*) is the number of occurrences of the pair of gray-level *i* and *j* at inter-pixel distance *δ* = (*DX, DY*). Assume the size of image is *L* × *L*, (*x*, *y*) are the coordinate of pixels, *x*, *y* = 0, 1, 2, . . ., *L* − 1, *f*(*x*, *y*) is the gray-level of pixels. Then the gray-level co-occurrence matrix of an image is as follows,

(5) $$p_{\delta }\left(i,j\right)=\left(\begin{array}{ccc} p_{\delta }(0,0) & \ldots & p_{\delta }(0,N-1)\\ \ldots & p_{\delta }(i,j) & \ldots \\ p_{\delta }(N-1,0) & \ldots & p_{\delta }(N-1,N-1) \end{array}\right)$$

Once the gray-level co-occurrence matrix is calculated, the texture features can be computed from it. In this paper, the texture features include the texture energy, texture entropy and texture contrast of the digital image of tobacco leaves. The formulae are as follows, the texture energy,

(6) $$E=\sum_{i=1}^{N}\sum_{i=1}^{N}{\left(p_{i,j}\right)}^{2}.$$

the texture entropy,

(7) $$H=\sum_{i=1}^{N}\sum_{i=1}^{N}p_{i,j}\;\text{log}\;p_{i,j}.$$

and the texture contrast,

(8) $$H=\sum_{i=1}^{N}\sum_{i=1}^{N}{\left(i-j\right)}^{2}p_{i,j}.$$

## 5. Theory of Fuzzy Set

The fuzzy set theory is the extension of conventional (crisp) set theory. It handles the concept of partial truth and the truth values between 1 (completely true) and 0 (completely false). Zadeh introduced it in 1965 as a mean to model the vagueness and ambiguity in complex systems [17–20]. Fuzzy theory became popular after 1980s and were used mainly by researchers in electronics and computer engineering. Nowadays, an immense number of studies are carried out using this method.

The fuzzy set theory, provide a rich and meaningful addition to standard logic. The mathematics generated by these theories is consistent, and the fuzzy logic may be a generalization of classic logic. The applications which may be generated from or adapted to the fuzzy logic are wide-ranging, and provide the opportunity for modeling of conditions which are inherently imprecisely defined, despite the concerns of classical logicians. Many systems may be modeled, simulated, and even replicated with the help of the fuzzy systems, not the least of which is human reasoning itself. Areas in which the fuzzy set theory has been successfully applied include artificial intelligence, auto control, information processing, economics, psychology, *etc*.

Simple fuzzy classification (SFC), fuzzy similarity method (FSM) and fuzzy comprehensive evaluation (FCE), are all subtitles of fuzzy synthetic evaluation (FSE) and have been used recently by a number of researchers in various areas [21,22]. Fuzzy evaluation methods process all the components according to predetermined weights and decrease the fuzziness by using membership functions, therefore sensitivity is quite high compared to other index evaluation techniques.

Let *X* is the set of objects, with elements noted as *x*. Thus, *X* = *x*. A fuzzy set *A* in *X* is characterized by a membership function *μ_A_*(*x*), which maps each point in *X* onto the real interval [0.0, 1.0]. As *μ_A_*(*x*) approaches 1.0, the membership function of *x* in *A* increases.

(9) $$A=\left\{x,\mu_{A}(x)|x\in X\right\},\mu_{A}(x):X\to [0,1].$$

The comprehensive fuzzy evaluation model is based on fuzzy set theory and the analytic hierarchical process which is developed by Saaty [23]. Saaty advocated the use of deductive systems approach in the analysis of complex decision problem. Zadeh define fuzzy logic underlying models of reasoning which are approximate rather than exact. The fuzzy comprehensive evaluation (FCE), which provides a high level confidence in decision making by the fuzzy logic, is a branch of artificial intelligence. It would classify or distinguish things by means of analyzing the fuzzy information as much as possible. Before making a decision, the fuzzy decision engine (FDE) in corporate different information. The information is usually a fuzzy quantization feature or the evaluations of some objects. According to the factors, a matrix is constructed and then a weight vector is assigned. Based on the matrix and the weight vector, the fuzzy decision engine can reach a final decision after a comprehensive evaluation.

As mentioned above, classification, quality evaluation or grading of tobacco leaves is a fuzzy problem, so the fuzzy comprehensive evaluation can be used for the automatic classification.

## 6. Automatic Classification of Tobacco Leaves

In this paper, nine features of the flue-cured tobacco leaves are extracted, which belong to three categories. The three classes of the flue-cured tobacco leaves are X1L, B4L and S1. The reason why we only choose three categories flue-cured tobacco leaves is that the process of classification, quality evaluation or grading need a lot of tobacco leaves which have been classified by experts. Getting all 40 categories is very difficult and very expensive. In fact, some categories of tobacco leaves are very rare. In this research, only 24 classified tobacco leaves are collected, and only eight categories have enough quantity (more than 100 pieces, we also need to divide them into two sets, training set and testing set) for the training of grading.

### 6.1. Mathematical Model

The comprehensive evaluation model gives a judgment to an object according to many factors in the fuzzy environment [24]. It gives a final judgment result. It is mainly made up by three essential factors, namely factor set, weight set and evaluation set.

Fuzzy comprehensive evaluation can be applied in three stages. Firstly, membership functions are determined. Then, using the membership functions, a fuzzy relationship matrix is formed. Lastly, the grading of tobacco leaves are given by fuzzy comprehensive evaluation.

In this paper, two-level fuzzy comprehensive evaluation is used for the classification of tobacco leaves. Firstly, we propose a mathematical model as follows.

1. The factor set

Assume that *U* = {*U*_1_, *U*_2_, . . ., *U_m_*} is a factor set, which is composed of *m* kinds of factors. It represents some attributes of tobacco leaves. Each factor *U_i_* can be separated into several sub-factors *U_ij_*(*i* = 1, 2, . . ., *m*; *j* = 1, 2, . . ., *n*), and the sub-factors *U_ij_* form some factor subsets *U_i_* = {*U*_*i*1_,*U*_*i*2_, . . .,*U_in_*}.

The factors which influence the classification of tobacco leaves are separated into three categories, *U* = {*U*_1_,*U*_2_,*U*_3_}. The sub-factors of every subsets are, *U*_1_ = {*U*_11_,*U*_12_,*U*_13_}, *U*_2_ = {*U*_21_,*U*_22_,*U*_23_}, *U*_3_ = {*U*_31_,*U*_32_,*U*_33_}.

*U*_1_ = *shape features*, *U*_2_ = *texture features*, *U*_3_ = *color features*, *U*_11_ = *area*, *U*_12_ = *perimeter*, *U*_13_ = *disfigurement*, *U*_21_ = *textureenergy*, *U*_22_ = *textureentropy*, *U*_23_ = *texturecontrast*, *U*_31_ = *variance of red*, *U*_32_ = *variance of green*, *U*_33_ = *variance of blue*.

2. The weight set

(1) Sub-factor weight set

A weight *a_i_*(*i* = 1, 2, . . ., *m*) represents the different influence on the decision-marking of every factors, thus the weight set of sub-factor is *A* = {*a*_1_,*a*_2_, . . ., *a_m_*}. According to the previous experiments, the most effective feature is the color features among the three features. We can effectively recognize the tobacco leaves according to the area of different color and the depth of color. The shape features are also very useful to the classification of tobacco leaves. While the texture features have less influence compares with the other two features. Therefore, we set *a*_1_ = 0.35, *a*_2_ = 0.2 and *a*_3_ = 0.45.

(2) Factors weight set

We arranged the factors weight according to the membership function of sub-factor *U_ij_*. The membership function comes from a great deal of experiments. *A*_1_ = {0.3, 0.4, 0.3}, *A*_2_ = {0.4, 0.3, 0.3}, *A*_3_ = {0.3, 0.3, 0.4}.

3. The evaluation set

The test tobacco leaves belong to three classes, so the evaluation sets are *V* = {*v*_1_ : *X*1*L*, *v*_2_ : *B*4*L*, *v*_3_ : *S*1}.

### 6.2. Computational Process

1. The sub-factor evaluation matrix.

A fuzzy set is fully defined by its membership function. How best to determine the membership function is the first question that has to be tackled. The approach adopted for acquiring the shape of any particular membership function is often dependent on the application. Because of the lack of a definite method for determining the membership function, we use artificial neural networks to determine it.

Back-propagation neural networks are probably the most well-known and widely applied of the neural networks today. In essence, the back-propagation net is a Perception with multiple layers, a different threshold function in the artificial neuron, and a more robust and capable learning rule.

**Figure 5.**
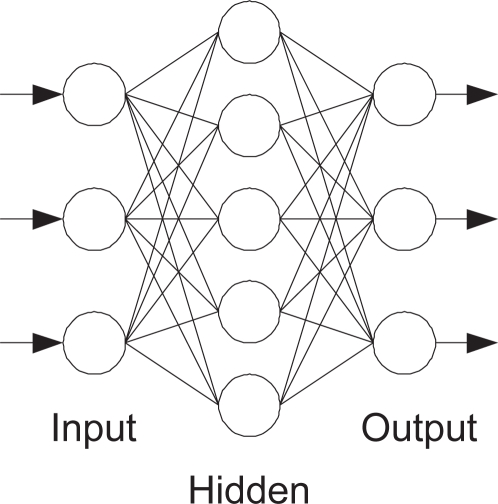
The structure of back-propagation neural networks.

Nine features are extracted from images of tobacco leaf. A back-propagation neural network is used to decide the membership of each feature in the fuzzy sets. The input layer of the BP neural network includes three neurons corresponding to the three features of a category of factors (shape, color or texture). The output layer includes three neurons corresponding to the membership function of three classes of tobacco leaves. A hidden layer is designed that includes five neurons. The structure of back-propagation neural network is shown as Figure 5.

In this paper, three features of a factors category (shape, color or texture) and the class of standard specimen tobacco leaves (The verdict of class is given by experts) are used in the training of neural network. For example, if a tobacco leaf belongs to X1L, then the output neuron corresponding to X1L is set to 1 and other two output neurons are set to 0. The training process is repeated until all the output is correct. Then we input three features of a factors category and get the membership of these features. The features of a factors category are normalized, $\sum_{j=1}^{n}r_{\mathrm{ij}}=1$. According to the membership *r_ij_*(*i* = 1, 2, . . ., *m*; *j* = 1, 2, . . ., *n*), we get a sub-factor evaluation matrix *R_i_*,

(10) $$R_{i}=\left(\begin{array}{cccc} R_{11} & R_{12} & \ldots & R_{1n}\\ R_{21} & R_{22} & \ldots & R_{2n}\\ \ldots & \ldots & \ldots & \ldots \\ R_{m1} & R_{m2} & \ldots & R_{\mathrm{mn}} \end{array}\right).$$

2. The one-level fuzzy comprehensive evaluation matrix B.

(11) $$B_{i}=A_{i}\cdot R_{i}.$$

(12) $$B={\left(B_{1},B_{2},\ldots ,B_{m}\right)}^{T}.$$

3. The two-level fuzzy comprehensive evaluation set *V*.

(13) V=A⋅B.

4. The evaluation set *V* and the class of the test tobacco leaves.

(14) $$V_{i}=V_{i}/\sum_{i=1}^{3}V_{i}$$

(15) $$\mu_{A}(x)=\max\left(V_{1},V_{2},V_{3}\right)$$

## 7. Experimental Results

In this research, 300 images of tobacco leaves are selected from the database, which include three classes of tobacco leaves—X1L, B4L and S1. Nine features are extracted from images of tobacco leaf, and these features are used for the learning of back-propagation neural network to get the membership of features in the fuzzy sets.

Three categories of factors (shape, color or texture) are extracted according to image processing and machine vision techniques. Experimental results of some tobacco leaves are shown in Tables 1–3.

Then, two group images of tobacco leaves are used in experiments. The first group includes 100 images, which comes from the images that have been trained by neural network. According to the results of the two-level fuzzy comprehensive evaluation, the accuracy rate of classification is 94%. The second group includes 50 images, which comes from the images that have been classified but have not been trained by neural network. After the two-level fuzzy comprehensive evaluation, the accuracy rate of classification is 72%.

Following is an evaluation process of a tobacco leaf. The average values of features from the three classes of standard specimen tobacco leaves are shown in Table 4. Table 5 shows the features of a test tobacco leaf. The grades of membership are shown in Table 6. We get the sub-factor evaluation matrix *R_i_* according to the membership, then calculate *B_i_* = *A_i_* · *R_i_* and get the one-level fuzzy comprehensive evaluation matrix ***B***,

$$B={\left(B_{1},B_{2},\ldots ,B_{m}\right)}^{T}=\left(\begin{array}{ccc} 0.23 & 0.57 & 0.2\\ 0.23 & 0.53 & 0.21\\ 0.26 & 0.54 & 0.2 \end{array}\right).$$

After the two-level fuzzy comprehensive evaluation, we get the evaluation set *V*,

V=A⋅B=(0.2345,0.5575,0.202).

We normalize the evaluation set, *V* = {0.2359, 0.5609, 0.2032}. According to the two-level fuzzy comprehensive evaluation, the grade of membership of test tobacco leaf is 0.2359 to the X1L, 0.5609 to the B4L and 0.2032 to the S1. Therefore this test tobacco is evaluated to fall in the B4L class of flue-cured tobacco leaves.

## 8. Conclusions

This paper proposes an automatic grading method of tobacco leaves based on the digital image processing and the fuzzy sets theory. A neural network is used to decide the membership function of the features of tobacco leaves in the fuzzy sets. The aim of this paper is to find a feasible method to realize the automatic grading of tobacco leaves. The experimental results of the two-level fuzzy comprehensive evaluation show that the accuracy rate of classification is about 94% for the trained tobacco leaves, and the accuracy rate of the non-trained tobacco leaves is about 72%. Automatic grading of tobacco leaves is a very complex task. We think that 72% accuracy rate of the non-trained tobacco leaves is a challenging result. Even though this performance is not practically exciting, the experimental results show that fuzzy comprehensive evaluation is a viable way for the automatic classification or quality evaluation of tobacco leaves. In the future, more works will be done, *i.e.*, (1) the quality of standard specimen tobacco leaves should be improved, (2) more digital features will be extracted, which precisely express the properties of tobacco leaves, and (3) more categories of tobacco leaves will be dealt with.

## Figures and Tables

**Table 1.** Shape features of some tobacco leaves.

No.	Surface area (Number of pixels)	Surface perimeter (Number of pixels)	Disfigurement (%)
1	40502	1080	1.28
2	48607	1308	0.56
3	39775	1013	3.96
4	57862	1406	3.01
5	18515	544	4.74
6	25924	956	1.90
7	10803	289	2.91
8	14435	419	3.12
9	59146	1750	2.16
10	30747	727	9.47
11	26838	625	1.47

**Table 2.** Color features of some tobacco leaves (Dimensionless unit).

No.	Variance of red	Variance of green	Variance of blue
1	185.4286	150.7143	95.14286
2	185.5714	153	98.57143
3	183	154.3333	103
4	157.2857	123.1429	76.14286
5	162	132	87.14286
6	175	144.1667	94.33334
7	163.8571	129.1429	81
8	174	141	90.42857
9	132	102.6667	66.33334
10	157.5714	122.5714	77.71429
11	160	127.2857	82
12	167.2	136.6	88.4
13	162.8571	135.5714	88.85714
14	130.1667	100.3333	65.16666
15	141	110.4286	73.42857
16	129.2	102.6	69.4
17	120.625	94.25	65.125
18	114.5	91.75	67
19	119.7143	94.71429	68.28571
20	111.5714	91.42857	68.14286
21	170.4286	138.2857	89.42857
22	150.2857	116.1429	72.28571
23	142.7143	113.4286	75.42857
24	160.7143	126.4286	77.28571

**Table 3.** Texture features of some tobacco leaves (Dimensionless unit).

No.	Texture energy	Texture entropy	Texture contrast
1	47.85714	145	33.14286
2	45	150.2857	35.42857
3	51	135.8333	33.33333
4	44	144.5714	32.42857
5	57.28571	121.5714	31.28572
6	54	126.8333	27.83333
7	35	172.1429	41
8	48	143.8571	34
9	35.33333	158.6667	33.33333
10	38.28571	163.7143	39.85714
11	44.85714	146.5714	34.28571
12	61.6	110.4	27.4
13	71.57143	89.28571	25.42857
14	40	149.3333	32.33333
15	61.28571	106.5714	26.71428
16	69.2	85.4	21.8
17	52.25	121.25	27
18	64.75	94	24
19	46.42857	128.2857	25.57143
20	53.42857	113.8571	25.42857
21	42.57143	152.2857	35.57143
22	42.28571	153	37.71429
23	55.28571	120.4286	29.28572
24	39.14286	157	35.14286

**Table 4.** Features of three classes (X1L, B4L and S1) of the standard specimen tobacco leaves.

*U*_11_	*U*_12_	*U*_13_	*U*_21_	*U*_22_	*U*_23_	*U*_31_	*U*_32_	*U*_33_
2123	41937	0.011	0.48	−1.43	33.14	887	696	575
941	16478	0.036	0.71	−0.9	25.43	1027	826	593
2069	41242	0.08	0.42	−1.53	35.57	633	612	523

**Table 5.** Features of a test tobacco leaf.

*U*_11_	*U*_12_	*U*_13_	*U*_21_	*U*_22_	*U*_23_	*U*_31_	*U*_32_	*U*_33_
820	18236	0.038	0.72	−0.89	25.22	1032	853	698

**Table 6.** The grade of membership.

	*U*_11_	*U*_12_	*U*_13_	*U*_21_	*U*_22_	*U*_23_	*U*_31_	*U*_32_	*U*_33_
X1L	0.2	0.2	0.3	0.2	0.3	0.2	0.3	0.1	0.3
B4L	0.5	0.6	0.6	0.5	0.5	0.7	0.5	0.7	0.5
S1	0.3	0.2	0.1	0.3	0.2	0.1	0.2	0.2	0.2
